# Effect of carbohydrates on the adhesion of *Bordetella bronchiseptica* to the respiratory epithelium in rabbits

**DOI:** 10.1007/s11259-024-10307-1

**Published:** 2024-02-10

**Authors:** Pilar Patiño, Carolina Gallego, Nhora Martínez, Carlos Iregui, Alba Rey

**Affiliations:** 1 Pathobiology Group, Laboratory of Veterinary Pathology, Faculty of Veterinary Medicine and Zootechnics, Universidad Nacional de Colombia (UN), Bogotá D.C., Colombia; 2 Laboratory of Veterinary Pathology, Universidad de Ciencias Aplicadas y Ambientales, Bogotá D.C., Colombia; 3 Faculty of Agricultural Sciences, Veterinary Medicine Program, Fundación Universitaria Agraria de Colombia, Bogotá D.C., Colombia

**Keywords:** Carbohydrates, *Bordetella bronchiseptica*, Respiratory epithelium, Anti-adhesive therapy, Rabbits

## Abstract

This study proposes an ecological approach for preventing respiratory tract infections caused by *Bordetella bronchiseptica* in mammals using a mixture of carbohydrates. In an in vivo study, 51-day-old New Zealand rabbits were treated with a solution containing 1 × 10^7^ CFUs of *B. bronchiseptica* and 250 μg of one of the following carbohydrates: N acetylglucosamine (GlcNAc), N acetylgalactosamine (GalNAc), alpha methyl mannose (AmeMan), alpha methyl glucose (AmeGlc) and sialic acid (Neu5AC). Positive (*B. bronchiseptica*) and negative (Physiological Saline Solution (PSS)) controls were included. Animals treated with GlcNAc or AmeGlc showed no clinical signs of infection and exhibited a significant reduction (*p* < 0.05) in the severity of microscopic lesions evaluated in the nasal cavity and lung compared with the positive controls. Additionally, the presence of bacteria was not detected through microbiological isolation or PCR in the lungs of animals treated with these sugars. Use of a mixture of GlcNAc and AmeGlc resulted in greater inhibition of microscopic lesions, with a significant reduction (*p* < 0.05) in the severity of these lesions compared to the results obtained using individual sugars. Furthermore, the bacterium was not detected through microbiological isolation, Polymerase Chain Reaction (PCR) or indirect immunoperoxidase (IIP) in this group.

## Introduction

*Bordetella bronchiseptica* is a motile Gram-negative coccobacillus and is part of the normal flora of the upper respiratory tracts of various host animals. *B. bronchiseptica* is currently known to contribute to various respiratory syndromes affecting a wide range of mammals, including dogs (Bemis et al. 1977; Molina et al. 2006; Fastres et al. 2020), cats (Welsh 1996), pigs (Pedersen and Barfod 1981; Saade et al. 2020), cattle (Liu et al. 2009), horses (Garcia-Cantu et al. 2000) and rabbits (Deeb et al. 1990; Gallego et al. 2013; Wang et al. 2020; Nielsen et al. 2021). There have also been cases of respiratory illnesses associated with *B. bronchiseptica* in immunocompromised humans (Woolfrey and Moody 1991; Llombart et al. 2006; Rath et al. 2008; Galeziok et al. 2009; Wang et al. 2020; Rybolt et al. 2022).

*B. bronchiseptica* adheres extensively to the epithelial cells of the upper respiratory tract of its hosts and it is mostly detected on the apical portion of the ciliated cells; it can also be found in the cytoplasm of neutrophils and macrophages. It adheres to the surface of the respiratory cells through structures and substances on its surface that act as adhesins, such as filamentous hemagglutinin (FHA), fimbriae, adenylate cyclase, pertactin and flagella (Savelkoul et al. 1996; Sisti et al. 2002; Hibrand-Saint Oyant et al. 2005; Edwards et al. 2005; Edwards 2006; Gallego et al. 2013; Saade et al. 2020), the first three of which are credited with mediating adhesion to host cells via carbohydrate-lectin interactions. Adhesion is the first step of infection and is therefore a central event in disease development. Once bacteria adhere to the surface of epithelial cells, they evade the cleaning mechanism of the mucociliary system and trigger infection via the expression of various virulence factors (Mattoo and Cherry 2005; Saade et al. 2020).

On the other side, the development of antibiotic resistance is a common problem in the treatment of bacterial infections (Davies and Davies 2010; D’Costa et al. 2011), as in the case of *B. bronchiseptica*, which exhibits high resistance to various antibacterial agents (Wettstein and Frey 2004; Zhao et al. 2011a; Mazumder et al. 2012). Additionally, vaccines aimed at preventing infection by bacteria of the *Bordetella* genus have shown contradictory results, for example, despite the wide application of vaccination programs, mainly against *Bordetella pertussis* in humans, the prevalence of diseases caused by these organisms has increased (Mooi et al. 2001; Gilberg et al. 2002; Ellis et al. 2002; Gopinathan et al. 2007; Zepp et al. 2011; Schulz et al. 2014). *B. bronchiseptica* infections also remain a common veterinary problem (Foley et al. 2002; Di Martino et al. 2007; Zhao et al. 2011a, b). Therefore, the development of new strategies and tools for prevention and control of diseases caused by *B. bronchiseptica* are required. One alternative for preventing and, perhaps, treating bacterial infections is anti-adhesive therapy based on the use of carbohydrates (CHOs) that block specific recognition sites between bacterial adhesins and receptors on host cell surfaces localized on molecules and structures that include carbohydrate-recognizing lectins residues (Ahmed et al. 2022).

The first test of CHO anti-adhesive therapy was developed in 1979 and demonstrated that the simultaneous injection of *E. coli* type 1 fimbria with alpha methyl mannopyranoside into mouse bladders reduced the rate of bacteriuria (Aronson et al. 1979). In vivo tests involving respiratory infections with *Streptococcus pneumoniae* in rabbits showed that simultaneously intratracheal administration of the bacteria with lacto-N-neotetraose and sialylated derivatives and intranasal administration of these oligosaccharides 24 h post-infection reduced the incidence of pneumonia and bacteremia (Idänpään-Heikkilä et al. 1997). An in vitro study using a mixture of fructose, mannose and galactose reported the inhibition of adhesion of *Pseudomonas aeruginosa* to bronchial epithelial cells and the development of pneumonia in mice intratracheally instilled with a mixture of the bacteria and sugars (Bucior et al. 2013).

The respiratory mucosal surface of rabbits, similar to that of other species, is rich in glycoproteins (Brandley and Schnaar 1986; Schulte et al. 1990; Muramatsu 2000), moreover, some adhesins, including *B. bronchiseptica* FHA, possess properties of lectins and the ability to bind to CHOs; on this basis, this study demonstrates that inhibiting the adhesion of *B. bronchiseptica* to the respiratory epithelium of rabbits using N-acetyl glucosamine and alpha methyl glucoside, alone or as a mixture, avoids the clinical signs of the disease and prevents the development of lesions in the respiratory epithelium of the nasal cavity and the lung caused by this microorganism.

## Materials and methods

### *Bordetella bronchiseptica* strain

*B. bronchiseptica* was isolated from the turbinates and tracheae of rabbits displaying signs of rhinitis and pneumonia from farms in Sabana de Bogotá, Colombia. The bacterium was grown in BHI agar for 24 h at 37 °C, and tests were conducted for differentiation (Gram staining, oxidase, catalase and urease tests). Molecular identification was performed via amplification of the gene encoding the ribosomal 16S rDNA subunit using the following primer pair: (27f) 5'-AGAGTTTGATCMTGGCTCAG- 3' and (1942R) 5'-TACGGYTACCTTGTTACGACTT-3', followed by sequencing, which showed 99.4% similarity with the reference strain *Bordetella bronchiseptica* RB50.

### Sugars/Carbohydrates

Five CHOs were utilized in this study: N acetylglucosamine (GlcNAc), N acetylgalactosamine (GalNAc), alpha methyl mannose (AmeMan), alpha methyl glucose (AmeGlc) and sialic acid (Neu5AC) (Vector laboratories®, Burlingame, California, USA). The selection of these sugars was based on experiments using in vitro cultures of nasal cavities from rabbit fetuses, in which previous culture experiments showed that LCA (*Lens culinaris*), PNA (*Arachis hypogaea*), DBA (*Dolichos biflorus*), RCA_120_ lectin (*Ricinus communis*) and WGA (*wheat germ agglutinin*) significantly (*p* < 0.05) inhibited the adhesion of the bacteria to the apex of the ciliated epithelium (Carrillo et al. 2015). These lectins recognize one or more of these CHOs: GlcNAc, GalNAc, Neu5AC, galactose, mannose and glucose. Once the test results for the individual sugars were obtained, a mixture of the sugars that significantly inhibited microscopic injuries caused by *B. bronchiseptica* was used*.*

### Animals

The experiments used a total of 42 clinically healthy New Zealand White rabbits that were 36 days old and were microbiologically negative for *B. bronchiseptica* and *Pasteurella multocida*. The animals underwent an adjustment period of 15 days in the laboratory, and at 51 days of age, they were randomized into 14 treatment groups of three rabbits per group (Table 1 and 2).

**Table 1:** Test solutions administered to each treatment group of rabbits (*n* = 3)

Rabbit group	Test solutions	Dosage
1	*B. bronchiseptica* + GlcNAc	2 × 10^6^ CFU of *B. bronchiseptica* + 250 µg of carbohydrate/ 400 µL of PSS
2	*B. bronchiseptica* + AmeGlc	
3	*B. bronchiseptica* + AmeMan	
4	*B. bronchiseptica* + GalNAc	
5	*B. bronchiseptica* + Neu5AC	
6	GlcNAc	250 µg of carbohydrate/400 µL of PSS
7	GalNAc	
8	AmeMan	
9	AmeGlc	
10	Neu5AC	
11	*B. bronchiseptica* (Positive control)	2 × 10^6^ CFU of *B. bronchiseptica*/ 400 µL of PSS
12	PSS (Negative control)	400 µL of PSS

*CFU* Colony forming unit, *PSS* Physiological saline solution. Each animal received a total of 400 µL of test solution: 200 µL administered intranasally (IN) and another 200 µL intratracheally (IT) simultaneously

**Table 2:** Test solutions administered to rabbits treated with a mixture of sugars (*n* = 3)

Rabbit group	Test solutions	Dosage
13	*B. bronchiseptica* + Mixture of GlcNAc + AmeGlc	2 × 10^6^ CFU of *B. bronchiseptica* + a mixture (1:1) of the sugars at a concentration of 250 µg/ 400 µL of PSS
14	Mixture of GlcNAc + AmeGlc (control group)	mixture of the sugars at a concentration of 250 µg/400 µL of PSS

*CFU* Colony forming unit, *PSS* Physiological saline solution. Each animal received a total of 400 µL of test solution: 200 µL administered intranasally (IN) and another 200 µL intratracheally (IT) simultaneously

The administration procedure of the experimental solution as well as for euthanasia was similar. The animals were pre-anesthetized with acepromazine at 0.5 mg/kg SCT, xylazine at 5 mg/kg IM and ketamine at 35 mg/kg IM.

All procedures were approved and authorized by the Bioethics Committee of the Faculty of Veterinary Medicine and Animal Science, National University of Colombia (Act 006/2010).

### Test solutions and experimental design

The test solutions included a mixture of 200 µL of *B. bronchiseptica* (at a concentration of 1 × 10^7^ CFU/mL in PSS, resulting in a final concentration of 2 × 10^6^ CFU of *B. brochiseptica*) combined with 200 µL of each sugar (at a concentration of 250 µg/200 µL in PSS, giving a final concentration of 250 µg of carbohydrate) as indicated in Table 1. Later a similar solution of *B. bronchiseptica* mixed with the two sugars (1:1) that obtained significant results was used at a concentration of 250 µg/200 µL in PSS, (resulting in a final concentration 250 ug of carbohydrate) as detailed in Table 2. These solutions were incubated for 15 min at room temperature.

Each animal received a total of 400 µL of each test solution: 200 µL administered intranasally (IN) and another 200 µL intratracheally (IT) simultaneously, as described in Table 1 and 2.

The animals were examined every 4 h beginning at the time of instillation, and the presentation of the following clinical signs was evaluated: hypothermia, cyanosis of the mucous membranes and ears, dyspnea (orthopnea, rapid shallow breathing at rest) and death. At 72 h post-instillation, the rabbits were euthanized with Euthanex® at a dose of 1 mL/5 kg intracardially.

### Microbiological re-isolation and PCR

For microbiological re-isolation, samples from lungs were collected for standard microbiological and PCR techniques. Re-isolation was performed using swabs of the cranial lobe of the lung, which were cultured on BHI agar at 37 °C for 24 h for subsequent differentiation assays (Gram staining, oxidase test, catalase test).

DNA extraction was carried out in lung tissue using the commercially available ZR Genomic DNA™ Tissue Miniprep kit (Zymo Research Corp., California, U.S.A.). For the identification of *B. bronchiseptica* via PCR, the following primer sequences targeting the gene encoding filamentous hemagglutinin were used: (F) 5´-ATGACTGACGCAACGAACCGTTTCC-3´ and (R) 5´-GCGTTCTCGCCGGGCTCAGAAACTG-3´.

### Tissue processing

After the animals were sacrificed, the heads were removed, and two transverse sections, each approximately 0.5 cm thick, were obtained from the nasal cavity, starting at the first premolar. The rib cage was removed, and the entire respiratory system was separated from the larynx and infused with 3.7% formaldehyde via the trachea, under pressure from a 20 cm column of water, to achieve complete fixation and further expansion of the lung tissue. Sagittal sections of all lung lobes were obtained. Turbinate samples and full lungs were fixed in 3.7% formalin buffer for 24 h at 4 °C. The sections of the nasal cavity were then subjected to a process of decalcification using a 10% solution of EDTA disodium salt, pH 7, at 4 °C for 8 days. All tissues were processed via routine techniques using hematoxylin–eosin (H&E).

### Macroscopic evaluation of the lungs

All lungs were grossly evaluated and catalogued as apparently normal (AN) or according to the distribution of the lesions as having diffuse (D) or cranial lesions (CR).

### Microscopic evaluation of tissues

The tissues stained with H&E were evaluated in a blinded manner by a researcher using a light microscope with a 40 × objective. For each nasal cavity, a total of 6 sections were evaluated: 2 from the dorsal region, 2 from the central region and 2 from the ventral region of the tissue. The evaluation criteria were migration of polymorphonuclear neutrophils into the respiratory epithelium, loss of cilia and presence of bacteria. Each of the changes was assigned a score according to its degree of severity, ranging from absent to severe (Table 3). For each nasal septum, scores were obtained for each lesion in each of the fields, and the average score for each lesion was calculated.

**Table 3:** Score according to degree of severity of each lesion or the labeling percentage of *B. bronchiseptica* identified by IIP technique on the epithelium of the nasal septum and lung

Grade of severity (H&E)	Percentage of identified *B. bronchiseptica* by IIP	Score
Absent	0%	0
Very mild	> 0–15%	1
Mild	> 15–30%	2
Mild-Moderate	> 30–45%	3
Moderate	> 45–60%	4
Moderate-Severe	> 60–75%	5
Severe	> 75%	6

*H&E* hematoxylin–eosin, *IIP* indirect immunoperoxidase

In the lung sections, each lobe was analyzed, and a score was assigned based on the degree of severity (Table 3) of the following lesions: septal thickening, the presence of detritus in the alveolar and/or bronchiolar lumen, the presence of bacteria and the presence of pneumonic foci. For each lung, the scores for each of the lobes and the average score for each lesion were calculated.

### Indirect immunoperoxidase

The indirect immunoperoxidase (IIP) technique was developed to label *B. bronchiseptica* and determine its location in the tissues of the animals treated with the mixture of sugars. A primary polyclonal antiserum antibody against *B. bronchiseptica* was produced in sheep using a 1:1 dilution, and IgG secondary anti-sheep antibodies produced in donkey (Sigma, Aldrich®) at a 1:500 dilution were added. The commercial Liquid DAB Substrate Kit (Invitrogen™) was used to reveal the antibody binding. The nasal septum and lung tissues were observed with a light microscope, and a score was assigned to each tissue according to the labeling percentage (extent) of the bacteria adhered to the epithelium, ranging from absent to severe (Table 3).

### Statistical analysis

The severity of lesions in the lung and nasal cavity, and the degree of labeling of *B. bronchiseptica* through IIP were rated from 0 to 6. The average scores for each group were evaluated via ANOVA to determine whether there were differences between treatments, which was followed by Dunnett's test to compare different treatments to the positive control (Zar 1998; Martínez et al. 2011). Differences were considered statistically significant at *p* < 0.05.

## Results

### Trials of individual sugars

#### Clinical signs

**Fig. 1:**
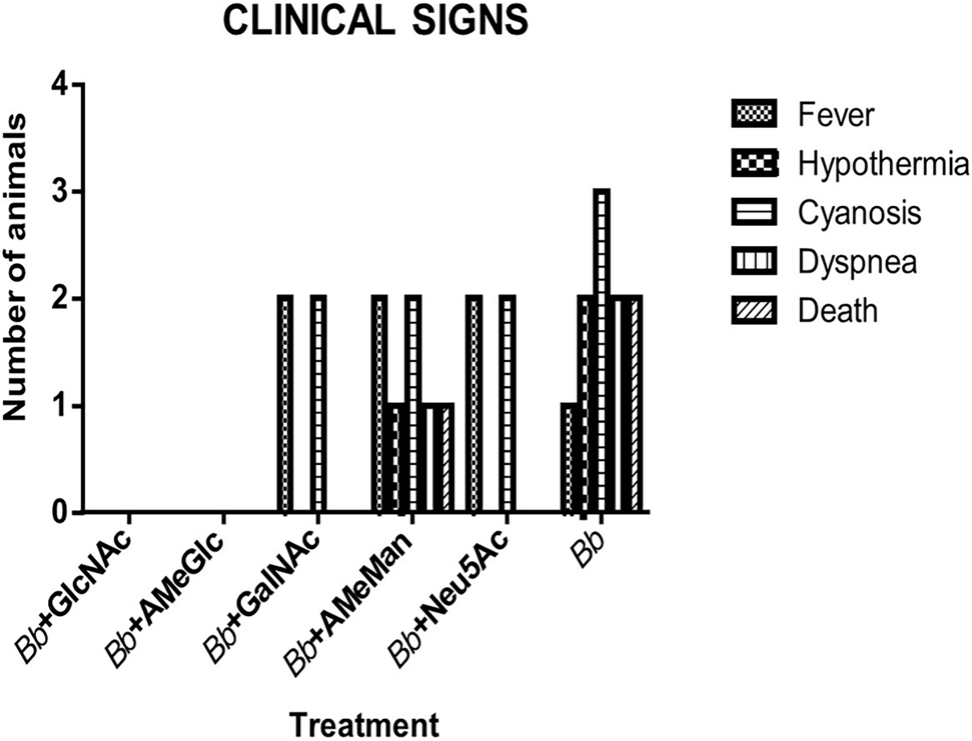
Number of rabbits with clinical signs in each experimental group treated with *B. bronchiseptica* and sugar (groups 1–5) or with *B. bronchiseptica* (group 11). *Bb*: *B. bronchiseptica*

None of the animals treated with *B. bronchiseptica* and GlcNAc (group 1) or AmeGlc (group 2) showed clinical signs. Furthermore, at least two of the three rabbits treated with *B. bronchiseptica* and AmeMan (group 3), GalNAc (group 4) or Neu5AC (group 5) evidenced clinical signs, predominantly fever and cyanosis of the mucous membranes and ears. Additionally, one individual treated with *B. bronchiseptica* + AmeMan (group 3) died from infection. The animals in the positive control group (group 11) showed greater severity of clinical signs: two of the three animals died approximately 24 h post-inoculation. No animal in the control CHO groups (groups 6–10) or the negative control group (group 12) exhibited clinical signs. Figure 1 shows the number of rabbits with clinical signs in the experimental groups treated with *B. bronchiseptica* (group 11) or with *B. bronchiseptica* and sugar (groups 1–5).

#### Microbiological re-isolation and PCR

PCR and conventional microbiological isolation techniques detected *B. bronchiseptica* only in the lungs of the animals in groups 3, 4, 5 and 11, which developed clinical signs.

#### Gross evaluation of the lungs

**Fig. 2:**
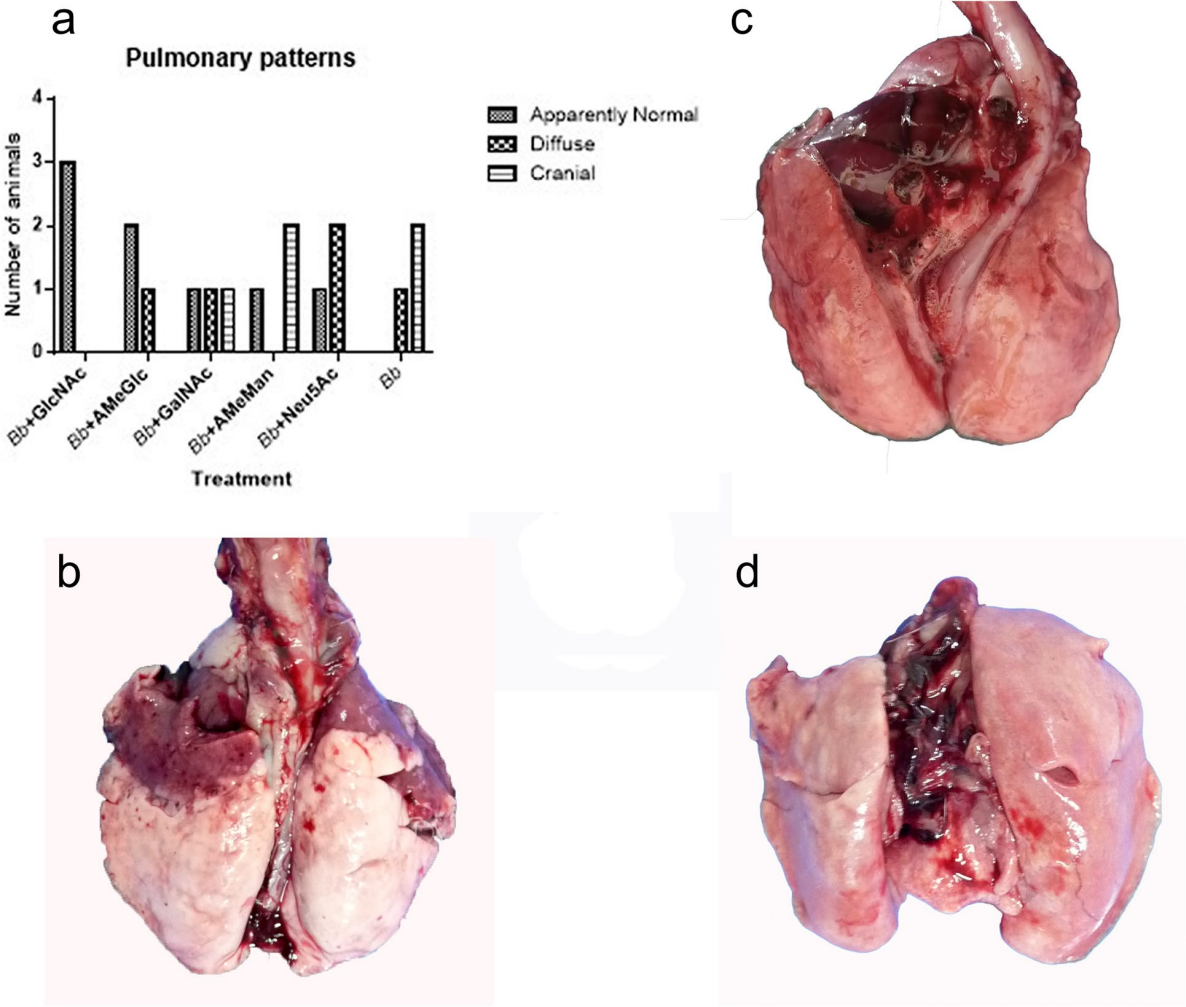
**a** Number of rabbits with the indicated patterns of pneumonia in each experimental group treated with *B. bronchiseptica* and sugar (groups 1–5) or with *B. bronchiseptica* (group 11 = positive control). **b** Lungs of a rabbit from the positive control group, note the cranioventral (darker regions) distribution of the bronchopneumonic lesions. **c** Lungs with a normal appearance from a rabbit treated with PSS (Group 12 = negative control). **d** Lungs with a normal appearance from a rabbit treated with *B. bronchiseptica* + GlcNAc (group 1). *Bb: B. bronchiseptica*

The three rabbits treated with *B. bronchiseptica* + GlcNAc (group 1) presented apparently normal lungs, and only one animal in the group treated with *B. bronchiseptica* + AmeGlc (group 2) developed a pattern of diffuse pneumonia. In the positive control group (group 11) and the other groups inoculated with bacteria and sugars, at least two rabbits exhibited some pattern of lung injury. None of the negative control animals (group 12) or CHO controls (groups 6 to 10) showed lung injury (Fig. 2).

#### Microscopic evaluation of the nasal cavity and lungs

##### Nasal cavity

**Fig. 3:**
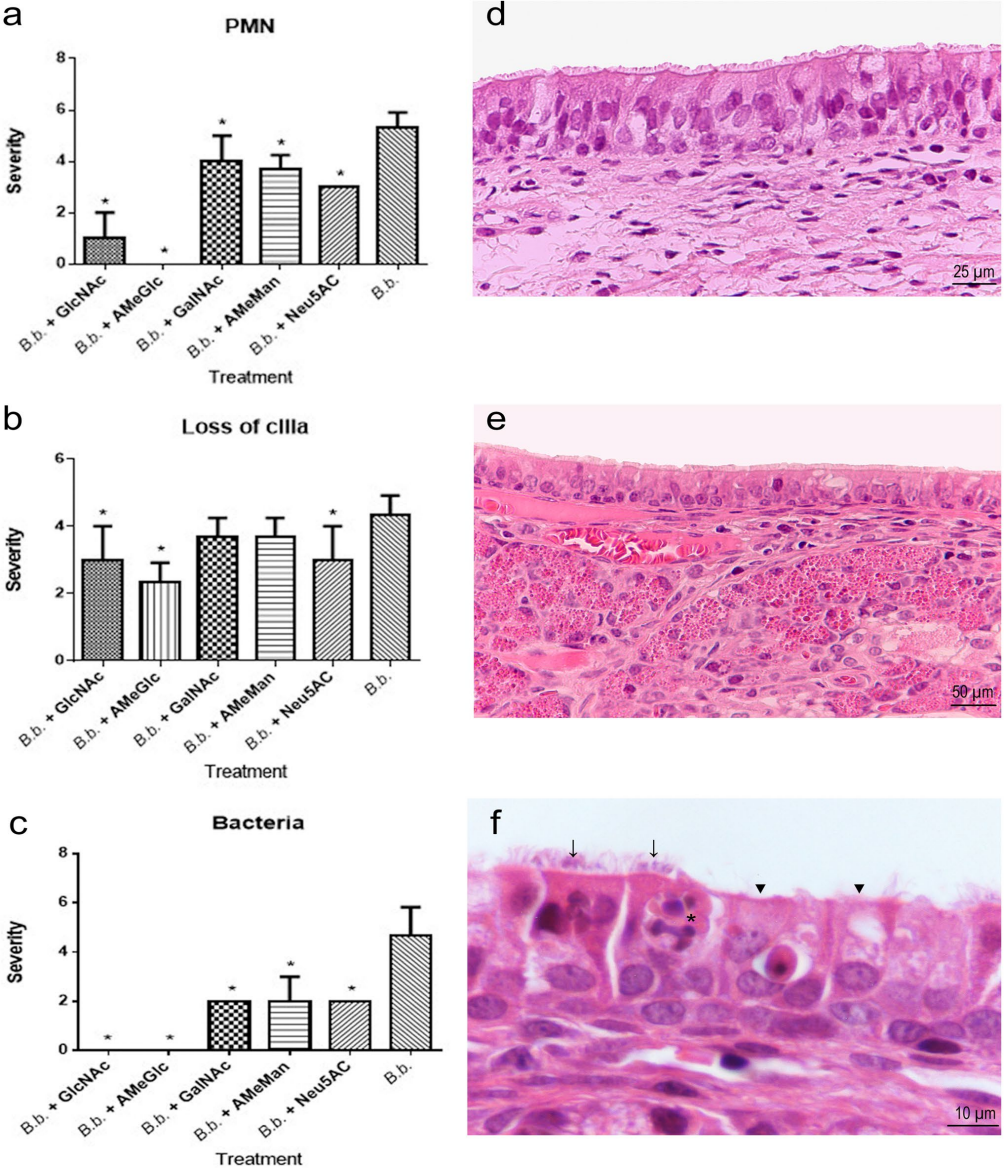
Severity of lesions based on microscopic evaluation of the respiratory epithelium in nasal cavities of rabbits in the groups treated with *B. bronchiseptica* + one CHO (groups 1–5) or *B. bronchiseptica* (group 11 = positive control). **a** Presence of PMNs. **b** Loss of cilia. **c** Severity of bacterial attachment to the apical surface of the respiratory epithelium. Histological section of the respiratory epithelium in nasal cavities of rabbits from (**d**) *B. bronchiseptica* + GlcNAc group (group 1) (H&E, 400X) and (**e**) Negative control (group 12) (H&E, 200X) show normal architecture. **f** Positive control (group 11) (H&E, 1000X) shows bacteria adhered between the cilia (arrows), loss of cilia (arrowheads) and PMNs infiltration between epithelial cells (asterisk); also, a dilatation of interepithelial spaces and disorganization of the epithelium is evident. *Bb: B. bronchiseptica*; *: significant difference (*p* < 0.05)

Only groups 1 (*B. bronchiseptica* + GlcNAc), 2 (*B. bronchiseptica* + AmeGlc) and 5 (*B. bronchiseptica* + Neu5AC) exhibited a lower severity (*p* < 0.05) of all lesions recorded in the nasal cavity compared with the positive control (group 11); in addition, the presence of bacteria was not observed in the first two groups, (Fig. 3a, b and c). Similarly, the negative control group (group 12) and the CHO controls (groups 6–10) were significantly different (*p* < 0.05) from the positive controls regarding all the microscopic lesions recorded in the nasal cavity. Figure 3d to 3f show histological sections of the rabbit nasal cavities with normal architecture and other with some lesions.

##### Lungs

**Fig. 4:**
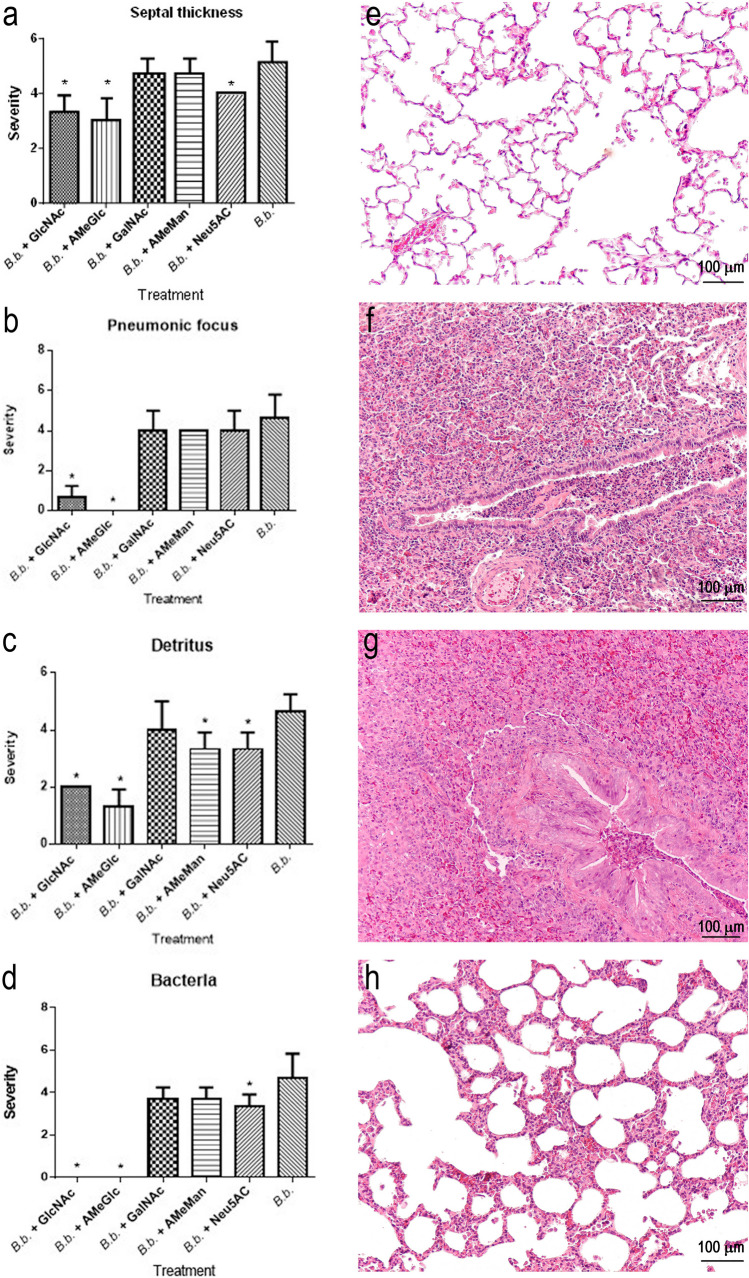
Severity of lesions based on microscopic evaluation of the lungs of rabbits in the groups treated with *B. bronchiseptica* + one CHO (groups 1–5) or *B. bronchiseptica* (group 11 = positive control). **a** Lung septal thickening. **b** Pneumonic foci. **c** Accumulation of detritus in the alveolar and bronchiolar lumen. **d** Bacterial accumulation in the alveoli. Histological sections of rabbit lungs from **e** Negative control (group 12) with the normal architecture retained (H&E, 100X). **f** Positive control (group 11) (H&E, 100X) and (**g**) *B. bronchiseptica* + GalNAc (group 4) (H&E, 100X), presenting a noticeable loss of architecture, with septal thickening and the presence of abundant inflammatory cells in the space of the bronchioles and severe pneumonic foci. **h** *B. bronchiseptica* + AmeGlc (group 2) showed a slight degree of septal thickening (H&E, 100X). *Bb*: *B. bronchiseptic,* *: significant difference (*p* < 0.05)

Among the animals treated with *B. bronchiseptica* plus CHO, only groups 1 (*B. bronchiseptica* + GlcNAc) and 2 (*B. bronchiseptica* + AmeGlc), compared with the positive control group (group 11), exhibited a lower severity (*p* < 0.05) of all lung lesions (Fig. 4a to 4d). The negative controls (group 12) and the CHO controls (groups 6–10) were also significantly different (*p* < 0.05) from the positive control. Figure 4e to 4h show histological sections of a lung with normal architecture and others with some lesions.

### Tests of sugar mixture: GlcNAc + AmeGlc

In each case in which clinical signs, gross lesions and the presence of microscopic lesions in both the nasal cavity and lungs of rabbits were significantly inhibited by the individual sugars GlcNAc and AmeGlc, a mixture of those sugars was used.

#### Clinical signs

None of the animals treated with *B. bronchiseptica* + the CHO mixture (group 13) or the mixture of CHOs (group 14) showed any clinical signs.

#### Microbiological re-isolation and PCR

*B. bronchiseptica* was not isolated from the lungs of any rabbits from groups 13 (*B. bronchiseptica* + CHO mixture) and 14 (CHO mixture), nor was identified by PCR technique.

#### Gross evaluation of the lungs

At necropsy, all the rabbits of group 13 (*B. bronchiseptica* + CHO mixture) and group 14 (mixed CHO) exhibited lungs that appeared normal.

#### Microscopic evaluation of the nasal cavity and lungs

##### Nasal cavity

**Fig. 5:**
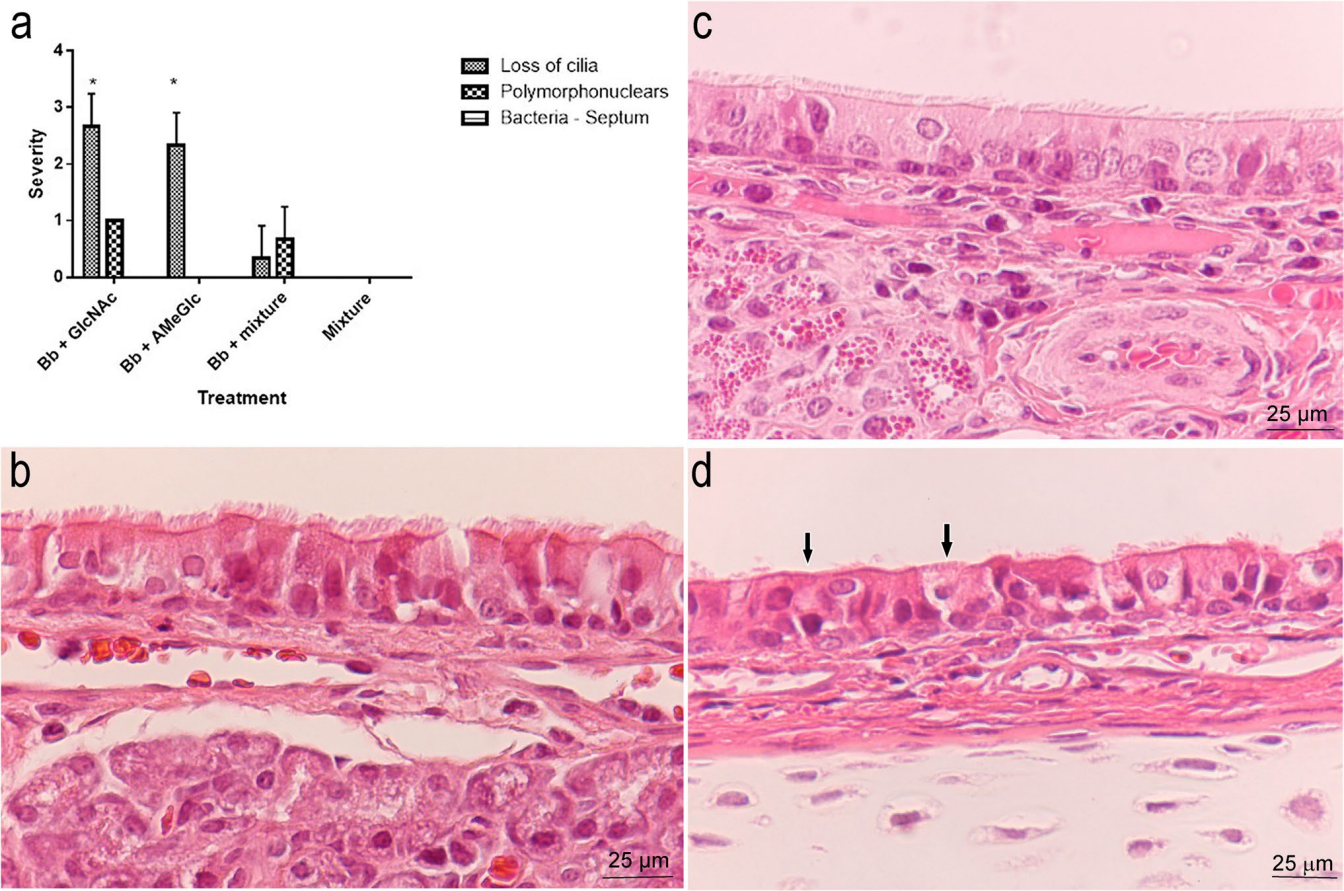
**a** Comparison of the severity of microscopic lesions in the nasal cavity between animals treated with *B. bronchiseptica* + individual CHOs (groups 1 and 2), animals treated with *B. bronchiseptica* + the CHO mixture (group 13) and animals treated with CHO mixture (group 14 = negative control). Histological section of the respiratory epithelium in the nasal cavities of rabbits from (**b**) *B. bronchiseptica* + the CHO mixture (group 13), (H&E, 400X) and (**c**) Negative control (group 14) (H&E, 400X), show normal architecture. **d** Positive control (group 11) (H&E, 400X) shows a notable loss of most cilia (arrows), with the remaining cilia being shorter than normal; disorganization of the epithelial cells is evident. *Bb: B. bronchiseptica*; *: significant difference (*p* < 0.05)

The respiratory epithelium of the nasal cavity of rabbits treated with *B. bronchiseptica* + the CHO mixture (group 13) exhibited a normal appearance (Fig. 5a); this group showed significantly less loss of cilia (*p* < 0.05) compared with the rabbits treated with *B. bronchiseptica* + individual sugars (groups 1 and 2) (Fig. 5b).

##### Lungs

**Fig. 6:**
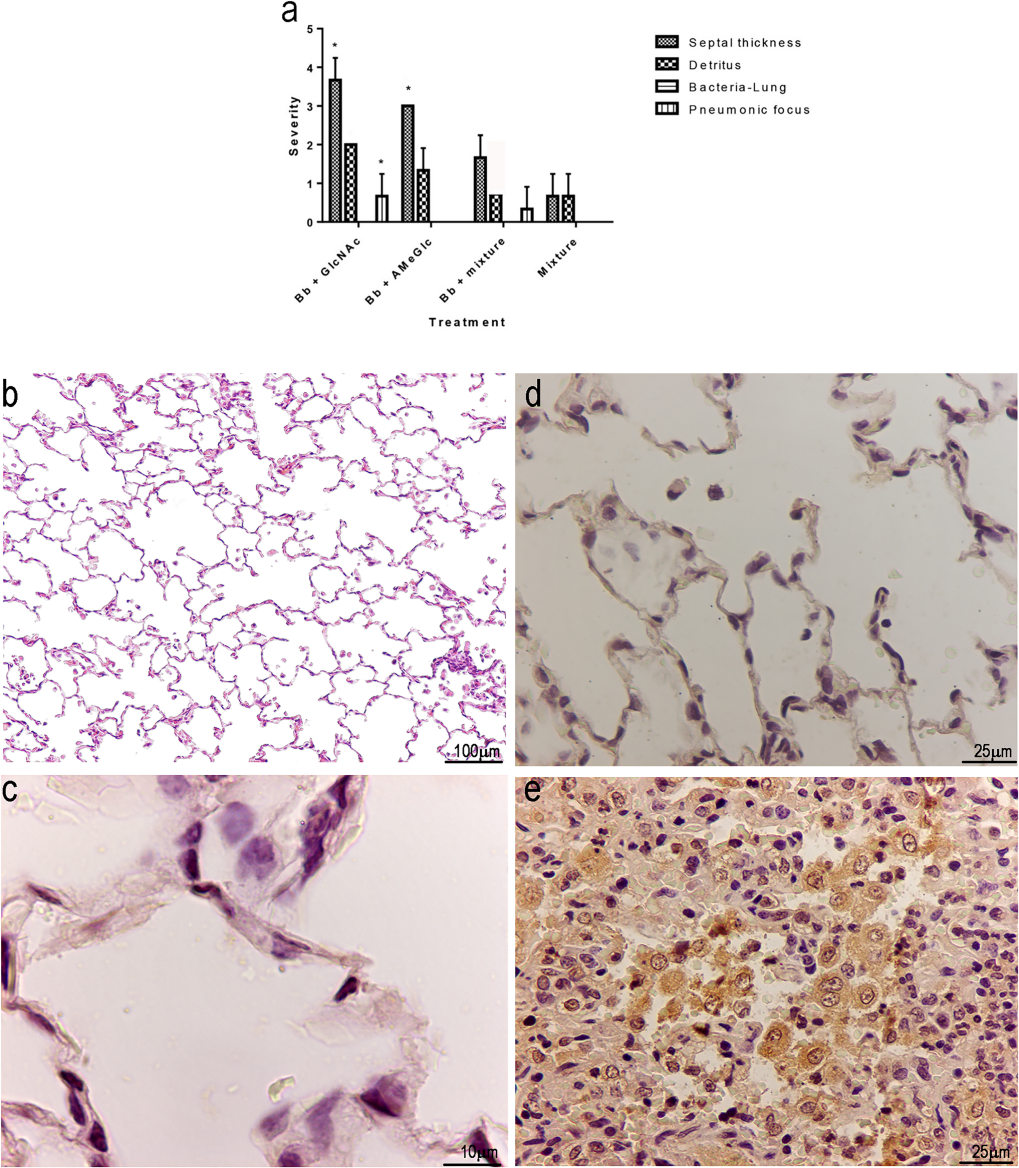
**a** Comparison of the severity of microscopic lesions in the lungs from animals treated with *B. bronchiseptica* + individual CHOs (groups 1 and 2) versus those treated with *B. bronchiseptica* + the mixture of GlcNAc and AmeGlc (group 13) and animals treated with CHO mixture (group 14 = negative control). Histological section of lung of a rabbit treated with *B. bronchiseptica* + the CHO mixture (group 13) showing (**b**) the normal architecture of the organ (H&E, 100X) and (**c**) staining indicative of the notable absence of *B. bronchiseptica* (IIP, 1000X). **d** Negative control (group 14) (IIP, 400X) where no specific labeling of the scant macrophages in the alveolar lumen is visible. **e** Positive control (group 11) (IIP, 400X) showing positive staining (brown color) indicating the presence of *B. bronchiseptica* both inside and outside of macrophages. *Bb*: *B. bronchiseptica,* *: significant difference (*p* < 0.05)

Significant differences (*p* < 0.05) in the severity of the thickening of the alveolar septa of the lungs were observed between group 13 (*B. bronchiseptica* + CHO mixture) and groups 1 (*B. bronchiseptica* + GlcNAc) and 2 (*B. bronchiseptica* + AmeGlc) and in the severity of the presence of pneumonic foci between groups 13 (*B. bronchiseptica* + CHO mixture) and 1 (*B. bronchiseptica* + GlcNAc) (Fig. 6a). The lungs of the rabbits in group 13 (*B. bronchiseptica* + CHO mixture) showed no microscopic changes indicative of a pneumonic process (Fig. 6b).

#### Indirect Immunoperoxidase

Moderate to severe positive IIP labeling of *B. bronchiseptica* was found in the nasal cavity and lungs of the rabbits in group 11 (positive control), while those of animals from group 13 (*B. bronchiseptica* + CHOs mixture) did not show positive staining (Fig. 6c to 6e) (*p* < 0.05).

## Discussion

Despite the use of various vaccines for the prevention of *B. bronchiseptica* infection, respiratory disease caused by this microorganism remains a common cause of significant economic losses in the livestock sector (Wabacha and Mulei 2000; Zhao et al. 2011a, b; Mazumder et al. 2012; Wang et al. 2020). In addition, in canines, *B. bronchiseptica* causes severe respiratory disease mostly represented by the kennel cough syndrome (Bhardwaj et al. 2013; Corona et al. 2013; Fastres et al. 2020); furthermore, the increasing resistance of the microorganism to antibiotics is another major concern (Mazumder et al. 2012; Nielsen et al. 2021). Hence, the effort to develop new ways to prevent and/or control these diseases deserves increasing attention. The present study demonstrated that treatment with the sugars GlcNAc and AmeGlc individually prevented the expression of clinical signs and gross lung lesions (except in one rabbit) and significantly inhibited the microscopic damages caused by *B. bronchiseptica* in the nasal cavities and lungs. Furthermore, the mixture of these two sugars even more significantly inhibited the microscopic lesions compared with the individual sugars.

Apparently both carbohydrates function as inhibitors of bacterial adhesion to nasal and pulmonary epithelial cells in rabbits, in turn preventing infection and the onset of respiratory disease in these animals. This prevention strategy is proposed as an ecological alternative that does not destroy the pathogen, perhaps preventing the development of *B. bronchiseptica* resistance, as can occur when antibiotics and vaccines are employed. Another purpose of this strategy is to avoid damage to the animal, particularly to the architecture of the epithelium of the respiratory tract and, thus, its functionality. Degenerative changes, necrosis and inflammation, that occur during *B. bronchiseptica* infections, as in other types of bacterial infections, lead to a cascade of events that are often more harmful to the host than the infection by itself (Anderson et al. 1991; Tetley 1993; Mogensen 2009). Among these events, we highlight over-regulation of the inflammatory response, which induces the accumulation of PMNs and the subsequent release of reactive oxygen species and proteolytic enzymes that cause tissue damage (Wright et al. 2010; Segel et al. 2011; Saade et al. 2020). *B. bronchiseptica* can also damage the cilia (Dugal et al. 1990; Anderton et al. 2004; Saade et al. 2020); together with the excessive release of mucus by goblet cells, this phenomenon significantly alters the efficacy of the mucociliary system. By preventing epithelial damage, it should theoretically be possible to preserve the structure and function of the mucociliary clearance machinery, thus aiding the host in expelling the bacteria from the body.

AmeGlc and GlcNAc could block the adhesion of bacteria to the epithelium via two mechanisms: by binding to lectin-type adhesins on the surface of *B. bronchiseptica* and/or by binding to lectins on the surface of host epithelial cells*.* Different adhesins expressed on the surface of *B. bronchiseptica* can act individually, collectively or synergistically; these adhesins include pertactin, fimbriae and filamentous hemagglutinin (FHA). FHA is one of the most important adhesins found in *B. bronchiseptica* and can mediate the adhesion of the bacteria to both ciliated and non-ciliated cells (Cotter et al. 1998; Nicholson et al. 2009; Saade et al. 2020). FHA has at least three binding sites on host cells: one site composed of carbohydrates that mediates adhesion to respiratory ciliated epithelial cells and macrophages (Tuomanen et al. 1988; Prasad et al. 1993; Saade et al. 2020); another site composed of glycine-arginine-aspartic acid (RGD) sequences that mediates leukocyte adhesion (Relman et al. 1990; Saade et al. 2020); and a glycosaminoglycan for binding to heparin, heparan sulfate and other sulfated carbohydrates (Hannah et al. 1994; Saade et al. 2020). It has also been reported that the adherence of *B. pertussis* FHA is largely mediated by lectin-carbohydrate interactions. However, there are contradictions regarding which carbohydrates might mediate the adhesion of both *B. pertussis* and *B. bronchiseptica*. Tuomanen et al. (1988) demonstrated that the adhesion of the *B. pertussis* FHA to ciliated human tracheal epithelial cells can be inhibited by molecules that contain lactose, such as galactose, galactose β1-4-glucose, complex lactosamines and by antibodies directed against molecules of galactose-bound GlcNAc (Gal B1-3GlcNAc [4–1-a-Fuc]). However, GalNAc had no significant effect on the inhibition of adhesion, similar to the findings of the present study in *B. bronchiseptica*. It would therefore be appropriate to evaluate whether simpler molecules, such as lactose and galactose, have an increased ability to inhibit the adherence of *B. bronchiseptica* to the epithelium of the respiratory tract of rabbits. Plotkin and Bemis (1984) achieved 100% inhibition of the adherence of *B. bronchiseptica* to hamster fibroblasts (HLF) by using GlcNAc, which is consistent with the results of the present study in rabbits; these authors were also able to reverse the adherence of the bacteria to HLF by 96% after 45 min of incubation with this CHO at a dose of 0.25 M.

However, the FHA does not appear to be the only adhesin from *B. bronchiseptica* that can bind to sugar residues on the respiratory epithelia of different species. For example, the *B. pertussis* fimbrial subunit fim2, which is also expressed by *B. bronchiseptica* (Savelkoul et al. 1996), binds to sulfated sugars such as heparan sulfate, chondroitin sulfate and dextran sulfate (Geuijen et al. 1996). Nevertheless, the specific binding domains for other fimbrial subunits from both *B. pertussis* and *B. bronchiseptica* have not yet been identified. Furthermore, some studies claim that *B. pertussis* pertactin, which is also expressed by *B. bronchiseptica*, mediates binding to the cell surface via RGD sequences (Leininger et al. 1991) in proline-rich regions (Williamson 1994) or leucine protein interactions (Emsley et al. 1994). It has also been proposed that the adenylate cyclase toxin (Hibrand-Saint Oyant et al. 2005) and the flagella (Savelkoul et al. 1996) contribute to the adherence of *B. bronchiseptica* to eukaryotic cells, although the mechanism of the binding of these structures remains unclear.

Additionally, the possibility that GlcNAc and AmeGlc or even glucose (which is the carbohydrate basis of these glycoconjugates) could also block the adherence of *B. bronchiseptica* by recognizing other bacterial structures cannot be excluded. More recent studies report that even when the expression of the major adhesins of *B. bronchiseptica* is blocked, its adhesive power is retained (Mattoo 2003 in Edwards 2006), and those authors propose lipopolysaccharide (LPS) as a possible factor that may contribute to the adherence and colonization in absence of these structures. Although there are no studies that concretely support this hypothesis in the case of *B. bronchiseptica*, research in *Salmonella enterica* (Bravo et al. 2011), *Pasteurella multocida* (Gallego et al. 2017) and *Helicobacter pylori* (Chang et al. 2011), suggests that this molecule plays an important role in the early steps of adherence to host epithelial cells. GlcNAc is part of the structure of the LPS of *B. bronchiseptica* (Preston et al. 2006), and incubation with this carbohydrate, as carried out in the present study, would be one of the mechanisms by which this sugar interferes with the binding of the bacterium to surface lectins of the rabbit respiratory tract.

The expression of FHA and, to a lesser degree, of the fimbriae, has been reported to be necessary for maximum biofilm formation by *B. bronchiseptica* (Irie et al. 2004). Thus, it might be speculated that blocking the binding of these adhesins with AmeGlc, GlcNAc or their mixture, as performed in this work, would not only inhibit the bacterial adhesion to their respective cellular receptors but also interfere with the biofilm formation. Still another mechanism by which AmeGlc and GlcNAc might have exerted their anti-adhesive activity in this study is by promoting bacterial agglutination or aggregation. Bacterial aggregation has been induced in other Gram-positive and Gram-negative species by a mix of simple carbohydrates such as fucose, mannose and galactose, that unlike the clusters formed in biofilms, those formed by the addition of exogenous carbohydrates are not stable and rapidly disperse (Bucior et al. 2013). Therefore, it is possible that the sugars used in this study promote bacterial aggregation, forming clusters through a similar process to the one that occurs during biofilm formation, but which is not sufficiently strong to promote biofilm formation, whereas it is sufficient to inhibit the adherence of bacteria to the epithelium and facilitate the removal of the pathogen by the mucociliary clearance system of the host.

Results of our group demonstrated that the previous incubation of *P. multocida* with individual GlcNAc, AmeGlc and AmeMan and a mixture of them, significantly inhibited the adherence of the bacterium to the respiratory epithelium of rabbits in an in vivo model. Equally so, it prevented the expression of clinical signs, microscopic and macroscopic changes in the nasal septa and lungs of rabbits experimentally exposed to the microorganisms (Gallego et al. 2021).

This work demonstrates that the anti-adhesive activity of the mixture of GlcNAc and AmeGlc appears to have at least an additive effect, when compared with the results obtained using the two CHOs individually. In turn, the inhibitory effect of the CHO mixture would block a greater diversity of bacterial adhesion sites, which eventually may indirectly indicate the existence of different adhesion structures with different compositions on the surface of the microorganism. It should be noted that therapeutic substances such as the CHOs studied here, in addition to presenting obvious environmental and economic benefits, exhibit other properties, such as a low probability that microorganisms will eventually develop resistance against them (Sharon 2006; Gallego et al. 2021).

In conclusion, GlcNAc, AmeGlc and, to a greater extent, a mixture of the two sugars significantly inhibited the adherence of *B. bronchiseptica* to the apical surface of the respiratory epithelia of nasal cavity and inhibited the colonization of the bacteria in the rabbit lungs, through which the establishment of infection and clinical manifestations and macro- and microscopic lesions were prevented. Subsequent studies must aim to develop more stable glycoconjugates with more prolonged effects that can be directly applied to animals and to evaluate their therapeutic capacity for treating *B. bronchiseptica* infections*.*

## Data Availability

The datasets generated and analyzed during the current study are available from the corresponding author on reasonable request.
